# MiR-146b inhibits autophagy in prostate cancer by targeting the PTEN/Akt/mTOR signaling pathway

**DOI:** 10.18632/aging.101534

**Published:** 2018-08-27

**Authors:** Song Gao, Zhiying Zhao, Rong Wu, Lina Wu, Xin Tian, Zhenyong Zhang

**Affiliations:** 1 The Second Department of Clinical Oncology, Shengjing Hospital, China Medical University, Shenyang 110022, China; 2 School of Computer Science and Engineering, Northeastern University, Shenyang 110004, China

**Keywords:** prostate cancer, microRNA-146b, PTEN, autophagy, AKT/m-TOR signaling pathway

## Abstract

Prostate cancer (PCa) is considered as a common visceral cancer in males and the sixth major cause of cancer-related deaths in males worldwide. Significant diagnostic and therapeutic advances have been made in the past decades. However, an improved understanding of their molecular mechanism is still needed. In the present research, we first detected the expression of miR-146b by quantitative real-time PCR (qRT-PCR) and found that miR-146b expression was increased in PCa. Subsequently, we found that miR-146b play an important role in the viability and proliferation capacity of PCa cells functionally. To explore the mechanism, we performed western blot to examine the autophagy-related markers, and found that miR‑146b may inhibit autophagy in PCa cells via activation of PTEN/AKT/mTOR signaling pathway. Furthermore, we performed the dual luciferase reporter assay to clarify the relationship between miR-146b and PTEN. In conclusion, this study demonstrated that miR-146b inhibited autophagy in PCa by targeting the PTEN/Akt/mTOR signaling pathway, and it could be a potential candidate for application in the treatment of PCa.

## Introduction

Prostate cancer (PCa) is known as a big health problem all over the world due to its high incidence morbidity and mortality, and this type of cancer is the second most common cancer and the sixth major cause of cancer-related deaths in males [1–3]. Estimated new cancer cases and deaths in United States in 2018 are 164,690 and 29,430 respectively, and the death rate dropped 52% from 1993 to 2015 for prostate cancer [4]. Although the improvements in the diagnostic methods of PCa and many new therapeutic methods have caused a decrease in PCa-related deaths over the past thirty years, and for patients who developed metastatic disease in the United States, their five-year survival rate was only 29% [5]. An understanding of many important molecules in the invasion and metastasis of PCa is currently being formed, however, substantial gaps in our knowledge remain. Therefore, understanding the novel molecular mechanism of PCa is urgently needed.

MicroRNAs (miRNAs) are small, single-stranded non-coding RNAs, which are 19–22 nucleotides in length, and they are capable to modulate protein expression via the sequence-specific targeting mRNAs [6,7]. More and more related studies have found that many deregulated miRNAs play a critical role of tumor suppressors or oncogenes in different kinds of cancer cells including PCa cells, and these miRNAs participate many biological processes, including metastasis, apoptosis and proliferation [8,9]. Dysregulation of numerous miRNAs, such as miR-15b, miR-29c, and miR-129, has already been confirmed in PCa [10–12]. These studies suggest that miRNAs could serve as a class of new molecular biomarkers or therapeutic targets for PCa. Among these miRNAs, miR-146b was overexpressed in many kinds of cancers. A previous study showed that miR-146b was unregulated in colorectal cancer tissues compared with the non-tumorous tissues, and it was involved in promoting cancer cells growth, invasion and glycolysis [13]. However, the therapeutic significance and function of miR-146b in PCa is still unclear.

Autophagy is considered as a lysosomal-dependent degradation way in cells, and it can produce amino acids and fatty acids for cell reusing via the degradation of the injured organelle proteins under the stress conditions [14,15]. Autophagy can be promoted or inhibited by many different signaling pathways, such as the PTEN/AKT/mTOR pathway [16,17]. Accumulating emerging evidence implicates autophagy can facilitate autophagic cell death and have a role in the tumorigenesis [18–20]. However, the effect of autophagy in PCa is still unknown.

In our research, we demonstrated that miR-146b may be a potential clinical biomarker in PCa. The roles of miR-146b on cell viability and autophagy may be through regulation of the PTEN/AKT/mTOR signaling pathway. These results might give us great insights to the molecular mechanism of miR-146b-regulated autophagy, and miR-146b be a potential candidate for application in the treatment of PCa.

## RESULTS

### MiR-146b is highly expressed in PCa cell lines and tissues

**Figure 1:**
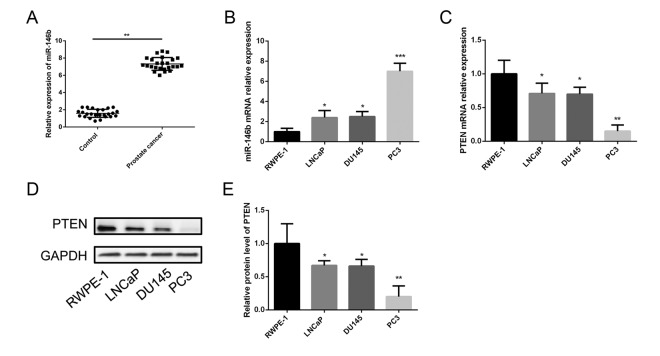
**Expression of miR-146b and PTEN in PCa tissues and cell lines.** (**A**) The qRT-PCR results showed that miR-146b mRNA was significantly upregulated in PCa tissues compared to the matched adjacent non-tumor tissues. (**B**) The qRT-PCR results showed that miR-146b mRNA was significantly upregulated in PCa cell lines. (**C**) The qRT-PCR results showed that PTEN mRNA was significantly downregulated in PCa cell lines. (**D**-**E**) The western blot results showed that protein level of PTEN was significantly downregulated in PCa cell lines. (*P < 0.05, **P < 0.01***P < 0.001).

In order to understand the clinical interrelation of the expression level of miR-146b in the PCa tissues, we analyzed 25 pairs of PCa tissue samples and non-tumor tissues by the method of qRT-PCR. The results showed that the expression of miR-146b was significantly upregulated in the PCa tumor tissues compared with the non-tumor tissues (control group) (Fig. 1A).

The expression level of miR-146b in LNCaP, DU145 and PC3 cells was detected using qRT-PCR. The results indicated that the relative expression levels of miR-146b were upregulated in the three prostate cancer cell lines significantly compared with RWPE-1 (Fig. 1B). It should be noted that miR-146b levels in the PC3 cells were obviously increased compared with those in the LNCaP and DU145 cells. Therefore, the PC3 cell line was selected for further investigation of the role of miR-146b in PCa. Furthermore, qRT-PCR and western blot were also performed to detect the mRNA and protein expression of PTEN in the four cell lines, and the results showed that PTEN levels in the PC3 cells were markedly decreased compared with those in the LNCaP and DU145 cells (Fig. 1C-E).

### MiR-146b influences the viability and proliferation in PCa cells

**Figure 2:**
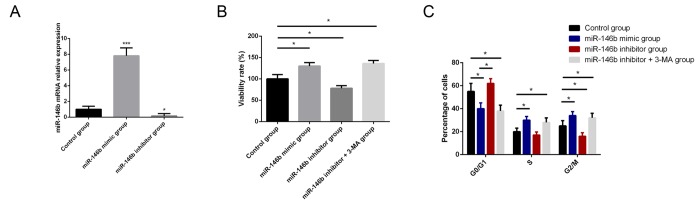
**MiR-146b influences the viability and proliferation of PCa cells.** (**A**) Transfection with miR-146b mimics and inhibitor in PC3 cells markedly increased and decrease the expression of miR-146b compared with control cells. (**B**) MTT analysis results showed that upregulation of miR-146b promoted PC3 cells survival while downregulation of miR-146b could suppress cell viability of PC3 cells. 3-MA, inhibitor of autophagy, could rescue the cell viability following transfection of miR-146b inhibitor. (**C**) The results of flow cytometry showed that upregulation of miR-146b could promote PC3 cells proliferation while downregulation of miR-146b could suppress cells proliferation. 3-MA could also rescue the PC3 cell proliferation capacity following transfection of miR-146b inhibitor. (*P < 0.05, ***P < 0.001).

To identify the biological function of miR-146b in PCa, PC3 cells were transfected with miR-146b mimic and miR-146b inhibitor to respectively increase and decrease its expression levels. Transfection with miR-146b mimics and inhibitor in PC3 cells markedly increased and decrease the expression of miR-146b compared with control cells (Fig. 2A). MTT results indicated that upregulation of expression levels of miR-146b could increase cell viability while downregulation of miR-146b could suppress cell viability of PC3 cells. We assumed that autophagy stimulated by miR-146b inhibitor was the cause of decreased viability of PC3 cell. We found that the 3-MA, inhibitor of autophagy, could rescue the cell viability following transfection of miR-146b inhibitor (Fig. 2B). After that, we also explored the regulatory role of miR-146b in proliferation of PC3 cells. The results indicated that upregulation of miR-146b could promote PC3 cells proliferation while downregulation of miR-146b could suppress cells proliferation. 3-MA could also rescue the PC3 cell proliferation capacity following transfection of miR-146b inhibitor (Fig. 2C).

### MiR-146b directly targets PTEN in PC3 cells

**Figure 3:**
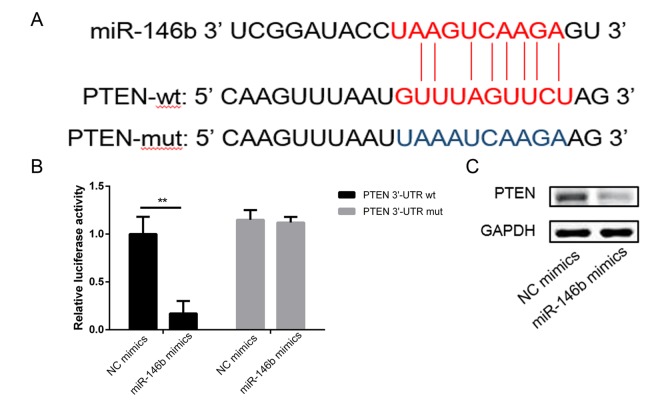
**MiR-146b directly targeted the 3’-UTR of PTEN mRNA.** (**A**) Schematic representation of the mature miR-146b sequence, putative miR-146b target site in the 3’-UTR of PTEN mRNA. (**B**) Overexpression of miR-146b markedly decreased the relative luciferase activity in the WT 3’-UTR of PTEN mRNA, while the mutated 3’-UTR of PTEN was insensitive to miR-146b overexpression. (**C**-**D**) MiR-146b mimic transfection decreased the expression of PTEN significantly. (**P < 0.01).

To investigate the related molecular mechanism by which miR-146b increased cells viability and promoted cells proliferation. miR-146b targets were predicted through bioinformatics analysis, and PTEN was shown to be a potential target of miR-146b (Figure 3A). Subsequently, the luciferase reporter assay indicated that co-transfection of miR-146b mimics and wild-type (WT) PTEN in PC3 cells led to a significant decrease in luciferase activity (Figure 3B). However, co-transfection of miR-146b mimic and PTEN mutant (MUT) did not lead to changes in luciferase activity compared to cells transfected with MUT PTEN alone. Western blot analysis corroborated this observation (Figure 3C). These findings confirmed that miR-146b targeted PTEN and inhibited its expression negatively.

### MiR-146b inhibited autophagy in PCa cells via PTEN/AKT/mTOR signaling pathway

**Figure 4:**
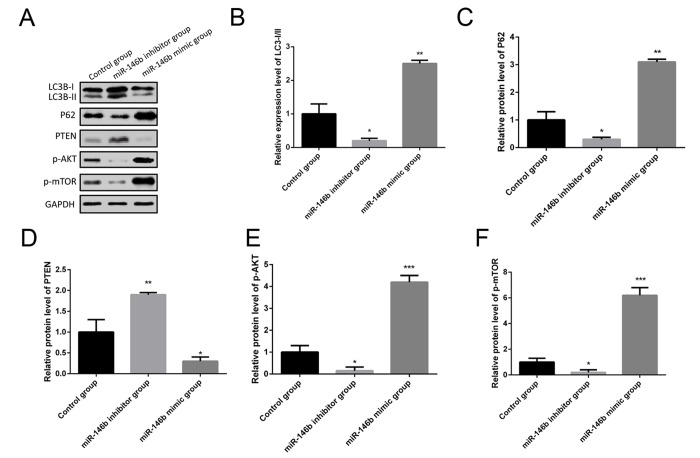
**MiR-146b inhibited autophagy via the PTEN/AKT/mTOR signaling pathway in PCa cells.** (**A**) Western blot results of the protein expression of LC3BI/II, P62, PTEN, p-AKT and p-mTOR in PC3 cells co-transfecting with miR-146b mimic, miR-146b inhibitor and negative control. (**B-F**) Quantification of the protein levels of LC3BI/II, P62, PTEN, p-AKT and p-mTOR. (*P < 0.05, **P < 0.01***P < 0.001).

More and more studies found that autophagy was identified as an important player in cancer tumorigenesis, so we hypothesized that miR-146b could promote PCa tumorigenesis by inhibiting autophagy. To experimentally verify this hypothesis, PC3 cells were co-transfected with miR-146b mimic, miR-146b inhibitor and negative control. Western blotting results showed the increased protein levels of P62 following miR-146b mimic transfection while decreased LC3-II to LC3-I conversion (Figure 4A-C). In addition, miR-146b mimic significantly decreased protein levels of PTEN while increased protein levels of p-AKT and p-mTOR (Figure 4A, D-F). In contrast, inhibition of miR-146b significantly decreased protein levels of P62, p-AKT and p-mTOR while increased protein levels of PTEN and LC3-II to LC3-I conversion (Figure 4). Taken together, these findings demonstrate that miR‑146b may inhibit autophagy cells via PTEN/AKT/mTOR signaling pathway in PCa.

## DISCUSSION

PCa is known as the most frequent non-cutaneous cancer and the second main cause of cancer-related mortality in all the countries [21]. The challenges for the treating the patients with PCa are because of the shortage of understanding the molecule mechanism of PCa. In this study, for the first time, we clarified the stimulative effect of miR-146b in the process of tumorigenesis in PCa, via targeting PTEN, and these findings also revealed that the fundamental mechanism through which miR-146b suppressed autophagy to inhibit the viability and proliferation of PCa cells involved the activation of AKT/mTOR pathway.

More and more researches have revealed that the vast majority of genomic products are transcribed in non-coding RNAs, including long non-coding RNAs and miRNAs [22–25]. miRNAs are short, non-coding RNAs (18-22 nucleotides) causing mRNA cleavage and then the degradation of mRNAs via binding to complementary 3’ untranslated region (UTR) of their targeted mRNA [26,27]. It is well-known that miRNAs are abundant and widespread, and they may play key roles in many basic cell processes, including cellular apoptosis, differentiation, growth, and migration, and dysregulated expression of miRNAs is a common feature in various cancers, including PCa [28–30]. miR-146b has been indicated to regulate cellular growth, metabolism and invasion, in many types of cancers [13,31]. In the current study, the results indicated that miR‑146b is highly expressed in PCa cell lines and tissues, and upregulation of miR-146b could promote PC3 cells proliferation.

Autophagy is a self-degradation system, which maintains normal cellular homeostasis, and autophagy-associated cell death is considered as a critical mechanism of non-apoptotic cell death [32,33]. In the previous years, emerging evidence showed that the activation or inhibition of autophagy may has key significance for the survival of tumor cells [34–37]. Autophagy was considered to be the main way to inhibit tumor cells proliferation [38,39]. A previous study found that highly upregulated in liver cancer (HULC) bound to Beclin-1, then inhibited Beclin-1 phosphorylation, and it could result in decreased autophagy via the modulation of the mTOR signaling pathway [40]. Furthermore, the inhibition of Serum- and glucocorticoid-induced protein kinase 1 (SGK1) could suppress PCa cells invasion and migration partially through autophagy-mediated inhibition of epithelial-mesenchymal transition (EMT) [41]. A recent study suggested that miR-200c-3p could regulate autophagy via upregulation of ER stress signaling [42]. Furthermore, miR-32 could directly target DAB2IP in PCa and suppress autophagy, and induce DAB2IP-deficient radioresistant human PCa cells [43]. MiR-212 could negatively modulate starvation induced autophagy in PCa cells through targeting sirtuin 1 (SIRT1) [44]. Moreover, it is emerging as a novel approach for blocking of autophagy in cancer cells via increasing the sensitivity in their treatment [45]. In our study, the results indicated that miR-146b is highly expressed in PCa tissues, and upregulation of miR146b may promote the viability and proliferation of PCa cells. Furthermore, we also found that the 3-MA, inhibitor of autophagy, could rescue the viability and the proliferation capacity of PC3 cells following transfection of miR-146b inhibitor. Furthermore, AKT/mTOR signaling pathway is also known as a key pathway regulating autophagy [46,47]. To identify the underlying mechanism, we speculate that miR-146b inhibited autophagy via the PTEN/AKT/mTOR signaling pathway in PCa cells. We performed western blot to examine the autophagy-related markers, and the results showed increased protein levels of P62 following miR-146b mimic transfection while decreased LC3-II to LC3-I conversion. In addition, miR-146b mimic significantly decreased protein levels of PTEN while increased protein levels of p-AKT and p-mTOR. In short, these results indicated that miR‑146b may inhibit autophagy PCa cells via PTEN/AKT/mTOR signaling pathway, and this may influence the viability and proliferation of PCa cells.

However, PTEN is not the only one that can be targeted by miR-146b. As we all know, a single miRNA may target numerous mRNAs while one mRNAs can also be targeted by multiple miRNAs [48]. Therefore, targeting PTEN by miR-146b is only one element in a complex regulatory network, and further studies which can identify other genes targeted by miR-146b or other miRNAs regulating PTEN will give us a deeper understanding about the molecular pathogenesis of OS.

In conclusion, this study demonstrated that miR-146b was upregulated in PCa tissues. Upregulation of miR-146b could promote PC3 cells proliferation while downregulation of miR-146b could suppress cells proliferation. These functions of miR-146b were due to the inhibition of PTEN and promotion of autophagy via AKT/mTOR signaling pathway in PCa cells. Together, our findings suggested that miR-146b could become a potential therapeutic target in PCa.

## MATERIALS AND METHODS

### Study samples

In all, 25 pairs of PCa tissues and matched adjacent non-tumor tissues were obtained between 2016 and 2017 from the Shengjing Hospital. All tissue samples were obtained after getting the written informed consent. The current study was also approved by the Ethics Review Board of China Medical University, Shenyang, China. PCa tissues were collected and immediately processed in the operating room and transported to the laboratory within 1 hour in appropriate culture and temperature condition. Following resection, we used the PBS to wash the tissues and they were immediately frozen in liquid nitrogen, and then they were stored at ‑80˚C. Then the expression of miR-146b in the PCa was compared with non-tumor tissues.

### Cell culture

Human PCa cell lines (PC3, LNCaP and DU145) and normal prostate epithelial cell line (RWPE-1) were obtained from the American Type Culture Collection (Manassas, VA, USA). Subsequently, the cells were cultured in RPMI-1640 medium (Gibco, Carlsbad, CA, USA) supplemented with 10% fetal bovine serum. All the cell lines were maintained at 37°C in a humidified atmosphere with 5% CO_2_.

### Quantitative real-time PCR (qRT-PCR)

First, we extracted the RNA from the cultured cells and tissues using TRIzol reagent (Invitrogen, USA). Reverse transcriptase was used to produce the first-strand complementary DNA (Aidlab, China) according to the manufacturer's instructions. miRNA Real-Time PCR Assay kit was used to detect the expression level of miR-146b (Aidlab, China). Furthermore, U6 was chosen to be internal control. The primers used were as follows: U6:5’-CTCGCTTCGGCAGCACA-3’ (forward), 5’-AACGCTTCACGAATTTGCGT-3’ (reverse); miR-146b:5’-TGACCCATCCTGGGCCTCAA-3’ (forward), 5’-CCAGTGGGCAAGATGTGGGCC-3’ (reverse); PTEN:5’- TGGATTCGACTTAGACTTGACCT -3’ (forward), 5’- GCGGTGTCATAATGTCTCTCAG -3’ (reverse).

### Cell transfection

Cells were all cultured in the medium for 24 h and seeded into 96-well plate for transfection. miR-146b mimic (20 nmol/L),miR-146b inhibitor (20 nmol/L) and negative control were all obtained from Genscript and were used according to the protocol. Then they were transfected into the cultured cells using LipofectamineTM 2000 reagent. The transfected cells were all collected and purified after 48 h incubation. The sequences of miR-146b mimic and inhibitor are as follows: miR-146b mimic: 5’-UGCCCUGUGGACUCAGUUCUGG-3’; miR-146b inhibitor: 5’-CCAGAACUGAGUCCAAGGGCA-3’.

### Luciferase reporter assay

In order to perform the target gene assays, a wild type (wt) 3’-UTR fragment of PTEN containing the putative miR-146b binding sequence was inserted into a pmirGlO Dual-luciferase miRNA Target Expression Vector, while mutant (mut) 3’-UTR was also cloned into the vector to generate PTEN-mutated-type contained mutated binding site. Cells at 60% confluence were co-transfected with PTEN-WT or PTEN-MUT and miR-146b mimics (20 nmol/L) using Lipofectamine™ 2000. After 48 hours, we used the dual Luciferase reporter assay system (Promega) to evaluate the activity.

### Western blot

All the cultured PC3 cells were lysed using RIPA buffer. We then used BCA method to detect the protein concentration. Subsequently, we used 10% SDS-PAGE gel to separate the protein lysates, and then they were transferred to PVDF membranes (Millipore, Billerica). Membranes were blocked and incubated with PTEN, LC3BI, LC3BII, and P62, p-AKT, p-mTOR antibody (1:5000, Abcam, USA) or GAPDH antibody (1:7000, Santa Cruz) at 4  °C overnight. Subsequently, the PVDF membranes were washed in TBST and incubated with horseradish peroxidase-conjugated secondary antibodies (1:5000, Abcam) at room temperature for 1 h. ECL Western blotting substrate (Pierce) was used for visualizing and detection.

### MTT assay

MTT assay was performed to detect the PC3 cells viability. We first seeded the different groups of PC3 cells into 96-well plate. Then, we added 20 μL MTT solution (5 mg/ml) was to each wells and incubated for 4 h at 37 °C. Then the absorbance at 492 nm was measured and the proliferation efficiency was examined. We repeated the experiments three times independently.

### Cell cycle analysis by flow cytometry

In order to perform the PC3 cell cycle analysis, we used the BD Cycletest™ Plus DNA Reagent kit to treat the cultured cells (BD Biosciences). We first gated out the cell debris and fixation artifacts. Subsequently, we quantified the cell populations at the G0/G1, S and G2/M phases using Modfit software.

### Statistical analysis

We used the GraphPad Prism 6.0 to conduct the experimental data (Graphpad Software Inc., San Diego, CA, USA). We used the two-tailed Student's t-test to detect the differences between two groups. Differences were considered to indicate significant difference at * *P* < 0.05, ** *P* < 0.01, *** *P* < 0.001 and **** *P* < 0.0001.
