# Correction: MiR-146b inhibits autophagy in prostate cancer through affecting PTEN/AKT/mTOR signaling pathway

**DOI:** 10.18632/aging.101780

**Published:** 2019-01-15

**Authors:** Song Gao, Zhiying Zhao, Rong Wu, Lina Wu, Xin Tian, Zhenyong Zhang

**Affiliations:** 1 The Second Department of Clinical Oncology, Shengjing Hospital, China Medical University, Shenyang 110022, China; 2 School of Computer Science and Engineering, Northeastern University, Shenyang 110004, China

Original article: Aging (Albany NY) 2018; 10: 2113-2121

**This article has been corrected:** The authors made two mistakes in text of Abstract and Discussion. The correct sentences are provided below. The authors declare that this correction does not change the results or conclusions of this paper. The authors sincerely apologize for this error.

## Abstract

To explore the mechanism, we performed western blot to examine the autophagy-related markers, and found that miR-146b may **inhibit** autophagy in PCa cells via activation of PTEN/AKT/mTOR signaling pathway.

## Discussion

In short, these results indicated that miR-146b may **inhibit** autophagy PCa cells via PTEN/AKT/mTOR signaling pathway, and this may influence the viability and proliferation of PCa cells.

